# MICAL2 Contributes to Gastric Cancer Cell Proliferation by Promoting YAP Dephosphorylation and Nuclear Translocation

**DOI:** 10.1155/2021/9955717

**Published:** 2021-10-05

**Authors:** Chenxiang Qi, Pengxiang Min, Qianwen Wang, Yueyuan Wang, Yixuan Song, Yujie Zhang, Maria Bibi, Jun Du

**Affiliations:** ^1^Department of Physiology, Nanjing Medical University, Nanjing, Jiangsu 211166, China; ^2^Key Laboratory of Cardio Vascular & Cerebrovascular Medicine, School of Pharmacy, Nanjing Medical University, Nanjing, Jiangsu 211166, China; ^3^The Laboratory Center for Basic Medical Sciences, Nanjing Medical University, Nanjing, Jiangsu 211166, China

## Abstract

Dynamic cytoskeletal rearrangements underlie the changes that occur during cell division in proliferating cells. MICAL2 has been reported to possess reactive oxygen species- (ROS-) generating properties and act as an important regulator of cytoskeletal dynamics. However, whether it plays a role in gastric cancer cell proliferation is not known. In the present study, we found that MICAL2 was highly expressed in gastric cancer tissues, and this high expression level was associated with carcinogenesis and poor overall survival in gastric cancer patients. The knockdown of MICAL2 led to cell cycle arrest in the S phase and attenuated cell proliferation. Concomitant with S-phase arrest, a decrease in CDK6 and cyclin D protein levels was observed. Furthermore, MICAL2 knockdown attenuated intracellular ROS generation, while MICAL2 overexpression led to a decrease in the p-YAP/YAP ratio and promoted YAP nuclear localization and cell proliferation, effects that were reversed by pretreatment with the ROS scavenger N-acetyl-L-cysteine (NAC) and SOD-mimetic drug tempol. We further found that MICAL2 induced Cdc42 activation, and activated Cdc42 mediated the effect of MICAL2 on YAP dephosphorylation and nuclear translocation. Collectively, our results showed that MICAL2 has a promotive effect on gastric cancer cell proliferation through ROS generation and Cdc42 activation, both of which independently contribute to YAP dephosphorylation and its nuclear translocation.

## 1. Introduction

Gastric cancer is one of the most commonly diagnosed malignancies worldwide [1, 2]. The malignancy of gastric cancer cells is strongly associated with their high proliferative ability. The Hippo pathway-related kinase cascade is a critical contributor to the balance among cell proliferation, differentiation, and apoptosis. Yes-associated protein (YAP), an effector of the Hippo signaling pathway, is a transcriptional coactivator for several key transcription factors that regulate the expression of a wide variety of cell proliferation-related genes [3–5]. A recent study revealed that YAP knockdown could inhibit tumor growth in a gastric cancer xenograft mouse model [6], suggesting that YAP has an important role in the development of gastric cancer and may be a novel target for attenuating gastric cancer cell proliferation.

The Hippo signaling pathway is highly associated with cell growth regulation. The core Hippo pathway signaling cascade is comprised of molecules such as MST1/2, SAV1, LATS1/2, YAP, and its paralog TAZ. Following the detection of an extracellular growth-inhibitory signal, a series of kinase cascade phosphorylation reactions are activated, leading to YAP phosphorylation. Cytoskeleton proteins then bind to phosphorylated YAP and sequester it in the cytoplasm, thereby reducing its nuclear activity and limiting cell proliferation. In contrast, unphosphorylated YAP can translocate into the nucleus, where it primarily interacts with the transcription factors TEAD1–4, thereby inducing the transcription of multiple oncogenes and promoting cell proliferation. Impaired Hippo signaling was reported to induce YAP- or TAZ-dependent oncogene addiction for cancer cells [7, 8]. Furthermore, YAP is localized to the nucleus in various types of human cancer, including breast, lung, and pancreatic cancers [9–11]. Although elevated YAP expression and its nuclear accumulation are known to be associated with poor disease-specific survival in gastric cancer patients [12], the precise molecular mechanisms underlying the regulatory effects of YAP in gastric carcinogenesis remain poorly understood.

A recent report indicated that YAP nuclear translocation could be induced *via* cytoskeleton tension and that this regulation required Rho GTPase activity and was independent of the canonical Hippo kinase cascade [13]. Rho GTPases are central regulators of actin reorganization, with Cdc42, Rac1, and Rho being the most prominent members. Cdc42 deficiency was reported to attenuate the Nwasp/stress fibers/YAP signaling pathway, leading to podocyte apoptosis [14]. Specifically, reduced Cdc42 levels lead to decreased F-actin content, which, in turn, frees up LATS1, allowing it to bind to and phosphorylate YAP, thereby inactivating it and reducing its nuclear accumulation, finally leading to attenuated cancer cell proliferation [15]. Although Cdc42 is known to exert its function by switching between an inactive GDP-bound state and an active GTP-bound form [16], the mechanisms involved in activating Cdc42 in gastric cancer cells are largely unknown.

Molecule interacting with CasL2 (MICAL2) is a microtubule-associated monooxygenase with a role in regulating cellular cytoskeletal dynamics through the induction of reactive oxygen species (ROS) production [17, 18]. MICAL2 is highly expressed in primary human epithelial tumors as well as in neoangiogenic endothelial cells in human solid tumors [19] and is thought to accelerate tumor progression [20–22]. MICAL2 has been reported to activate ERK1/2 signaling and promote vascular smooth muscle cell proliferation [23]. MICAL2-deficient breast cancer cells develop marked migration defects through the inhibition of P38/HSP27/cytoskeleton signaling [22]. We have recently shown that MICAL-L2, a member of the MICAL protein family, enhances the migratory ability of gastric cancer cells *via* regulating EGFR stability in a Cdc42-dependent manner. We further demonstrated that MICAL-L2 can activate Cdc42 [24]. As MICAL family members have a similar structure [25], we hypothesized that MICAL2 might promote YAP nuclear translocation *via* ROS generation and/or Cdc42 activation, thereby promoting gastric cancer cell proliferation. In the present study, we investigated whether MICAL2 regulates YAP activation and explored the underlying mechanism.

## 2. Materials and Methods

### 2.1. Ethics Statement

All immunohistochemistry assays with human tumors specimens were conducted under the institutional guidelines of Jiangsu Province.

### 2.2. Cell Culture

Human gastric cancer cell lines (BGC-823 and SGC-7901) and normal gastric epithelial cell line GES-1 were purchased from the Typical Culture Preservation Commission Cell Bank, Chinese Academy of Sciences (Shanghai, China). All cells were maintained in Dulbecco's modified Eagle's medium (DMEM; high glucose) (HyClone, Waltham, Massachusetts, USA) supplemented with 10% fetal bovine serum (FBS, Gibco, Carlsbad, California, USA), 100 U/ml penicillin, and 100 *μ*g/ml streptomycin (Invitrogen, Carlsbad, CA). The cells were cultured in a humid, constant temperature incubator (Thermo, Waltham, Massachusetts, USA) with 5% CO_2_ at 37°C. Cells were grown on coverslips for immunofluorescence staining and on 6-well plates (Thermo, Waltham, Massachusetts, USA) for protein extraction.

### 2.3. Plasmids and siRNAs

The pEGFP-N1 vector containing the full-length Cdc42-Q61L (CA) or Cdc42-T17N (DN) insert was kept in this laboratory. The empty vector control pcDNA-3.1-HA-C and the full-length human MICAL2 cDNA were purchased from YouBio (Hunan, China). The empty vector control pEGFP-N1 and the full-length human YAP1 cDNA were also purchased from YouBio (Hunan, China). The transfection procedure followed manufacturer's protocol, using Lipofectamine 2000 (Invitrogen, Carlsbad, California, USA).

The siRNAs were synthesized and purified by GenePharma (Shanghai, China). The transfection of MICAL2 siRNA or control siRNA with Lipofectamine 2000 was performed according to manufacturer's instruction. The siRNAs specifically targeting MICAL2 were as follows: #1, 5′-GAGAACGUGAACCAAGACATT-3′; #2, 5′-GCAUAGAUCUUGAGAACAUTT-3′; #3, 5′-GCAGCGACACGUGUUACUUTT-3′. The siRNAs specifically targeting YAP were as follows: #1, 5′-GCAUCUUCGACAGUCUUCUTT-3′; #2, 5′-GGUCAGAGAUACUUCUUATT-3′; #3, 5′-GGUAGCGCUUUGUAUGCAUTT-3′. After transfected with plasmid or siRNA for 24 h, the cells were cultured and then treated with N-acetyl-L-cysteine (NAC, scavenger of ROS) and tempol (SOD-mimetic drug) at the indicated time points.

### 2.4. CCK8 Assay

Cell viability was determined by the CCK-8 assay. In short, cells were seeded into 96-well plates at a density of 5 × 10^3^ cells per well and transfected with siRNA or plasmids according to the groups. After incubated for 0, 24, 48, and 72 h, 10 *μ*l of CCK-8 reagent (Bimake, Houston, Texas) was added per well. Then, the 96-well plate was incubated in the dark for 40 min and then measured at 450 nm using a microplate reader (Bio-Tek, Elx800, USA).

### 2.5. Flow Cytometry Analysis

Cell cycle analysis was performed by flow cytometry. In short, the cells were collected and fixed in 75% ethanol overnight at -20°C. The cells were then incubated with RNase A and propidium iodide staining solution at 37°C for 30 min in the dark. Subsequently, the stained cells were analyzed using a flow cytometer.

### 2.6. 5-Ethynyl-2-Deoxyuridine (EdU) Incorporation Assay

The cell proliferation was measured using EdU staining kit (RiboBio, Guangzhou, China) according to manufacturer's instructions. Three replicas were made for each group. In short, the cells were seeded on a 96-well plate (Thermo, Waltham, Massachusetts, USA) and cultured until 70% confluence was reached, and then, EdU reagent was added to the culture medium and incubated for 2 h. After labeled, the cells were washed 3 times with PBS and fixed with 4% paraformaldehyde. Then, the cells were incubated with 0.2% glycine and washed 3 times with 0.5% Triton X-100 in PBS. Finally, the cells were counterstained with DAPI and then imaged by a fluorescence microscope (Olympus BX 51, Tokyo, Japan) combined with an Olympus DP70 digital camera.

### 2.7. Western Blotting

The total protein sample was prepared using the cell lysate (Beyotime, Shanghai, China), and the protein concentration was measured using the BCA protein assay kit (Thermo, Waltham, Massachusetts, USA). By SDS-PAGE electrophoresis, an equal amount of cellular protein fragments were separated and transferred to a pure nitrocellulose membrane. Then, after blocking with 5% skimmed milk, the membrane was incubated with different specific primary antibodies overnight at 4°C and then incubated with secondary antibodies (Jackson, Lancaster, Pennsylvania, USA) for 1.5 h at room temperature for detection. The following antibodies were used: MICAL2 (ProteinTech, Wuhan, China), GAPDH (Bioworld, Nanjing, China), p-YAP (Cell Signaling, Danvers, MA and Affinity Biosciences, Cincinnati, OH, USA), YAP (Cell Signaling), Cdc42 (Cell Signaling), HA-tag (Cell Signaling), CDK2 (ProteinTech), CDK4 (ProteinTech), CDK6 (ProteinTech), cyclin D (Cell Signaling), cyclin E (Cell Signaling), cyclin H (Cell Signaling), NF-*κ*B (Cell Signaling), ERK (Cell Signaling), and p-ERK (Cell Signaling). After using an appropriate amount of secondary antibody at a dilution of 1 : 10000, ECL (FuDeBio, Hangzhou, China) reagent was used to detect bands, and positive bands were analyzed with Quantity One (Bio-Rad, Hercules, CA).

### 2.8. Cytoplasmic and Nuclear Protein Extraction

Cytoplasmic and nuclear proteins were extracted using the nuclear protein and cytoplasmic protein extraction kit (Beyotime, Shanghai, China). In short, the cells were collected and resuspended in cytoplasmic extractant A. The suspension was vortexed and incubated on ice for 15 min. Then, the cytoplasmic extractant B was added to the cell pellet. The pellet was resuspended and incubated on ice for 1 min. The pellet was vortexed again and centrifuged at 12,500 g for 5 min. The collected supernatant is the cytoplasmic protein extract. Then, the pellet was resuspended by adding a nuclear extraction reagent. After several times of vortex, the mixture was centrifuged at 12,000 rpm for 10 min. The collected supernatant was the nuclear protein extract.

### 2.9. Immunofluorescence Microscopy

The cells planted on the glass cover slip were washed 3 times with precooled PBS, then were fixed with 4% paraformaldehyde solution for 20 min. After treated with 0.2% Triton X-100 for 5 min and blocked with 1% BSA at room temperature for 1.5 h, the cells were incubated with specific primary antibody overnight. Then, Alexa-conjugated species-matched secondary antibody was used to incubate cells at room temperature for 1 h. DAPI (Southern Biotech, Birmingham, AL) staining was used to determine the position of nuclei. The immunofluorescence image was taken with Olympus DP70 digital camera and Olympus BX51 microscope (Olympus, Tokyo, Japan).

### 2.10. Pulldown Assay

Cdc42 activity was measured by pulldown assay as described previously [24]. The PAK-CRIB with GST tag is purified and extracted from BL21 bacteria, and the active Cdc42-GTP could be pulled down by PAK-CRIB magnetic beads. In short, the cells were lysed, and protein was collected in a new tube. 300 *μ*g of protein was mixed with PAK-CRIB precoupled beads by rotation at 4°C for 2 h. The beads were then washed, and the proteins bound to the beads were separated by SDS-PAGE gel electrophoresis. Active Cdc42 was determined by analysis of its specific bands by Western blotting.

### 2.11. Measurement of ROS

ROS measurement was carried out by kit as described previously [26]. For intracellular ROS staining, individual cells were inoculated in 6-well plate and treated with interfering strands, plasmids, or NAC (5 mM, 4 h) (Beyotime, Shanghai, China) and tempol (3 mM, 4 h) (MCE, New Jersey, USA), and then, 10 *μ*M 2′,7′-dichlorodihydrofluorescein diacetate (DCFH-DA) (Beyotime) or 10 *μ*M DHE (Beyotime) was used to stain the cells at 37°C for 30 min. The immunofluorescence image was taken with Olympus DP70 digital camera and Olympus BX51 microscope (Olympus, Tokyo, Japan). Green fluorescence (DCF) intensity was quantitated using the microscope with 488 nm excitation and 525 nm emission settings, respectively. Red fluorescence (HE) was measured with 300 nm excitation and 610 nm emission settings.

### 2.12. Immunohistochemistry

Gastric cancer tissue microarrays were purchased from Outdo Biotech (Shanghai, China). In this study, 30 samples of gastric adenocarcinoma and their corresponding precancerous tissue samples were subjected to immunohistological examination. After dewax and hydration, the microarray was incubated with 3% H_2_O_2_ for 30 min to inactivate endogenous peroxidase and then was incubated with citric acid antigen retrieval solution at 95°C for 20 min. The microarray was then blocked with goat serum for 2 h at room temperature. After incubated with MICAL2 antibody at 4°C overnight, the microarray incubated with species matched secondary antibodies (Maxim Biotechnologies, Fuzhou, China) for 2 h at room temperature. DAB solution was used to detect the expression of MICAL2. Sections were counterstained with hematoxylin. The photo was taken by Olympus BX51 microscope. By evaluating the percentage of the number of stained cells and the staining intensity on the staining scores of MICAL2, the immune response score (IRS) was evaluated as described previously [27].

### 2.13. Statistical Analysis

All experiments were repeated at least three times independently. Statistical differences between two groups were tested using Student's *t*-test. Comparisons among three or more groups were conducted using one-way ANOVA with a posttest to correct for multiple comparisons. The chi-squared test was used to evaluate the significance of correlation. *P* < 0.05 indicates statistical significance, and *P* < 0.01 and *P* < 0.001 indicate sufficiently statistical significance (two tailed). Error bars represent standard error of the mean. All calculations were performed with SPSS Version 20.0 (IBM Corp., Armonk, NY, USA).

## 3. Results

### 3.1. MICAL2 Is Highly Expressed in Human Gastric Cancer Samples and Is Associated with Poor Clinical Outcome

**Figure 1:**
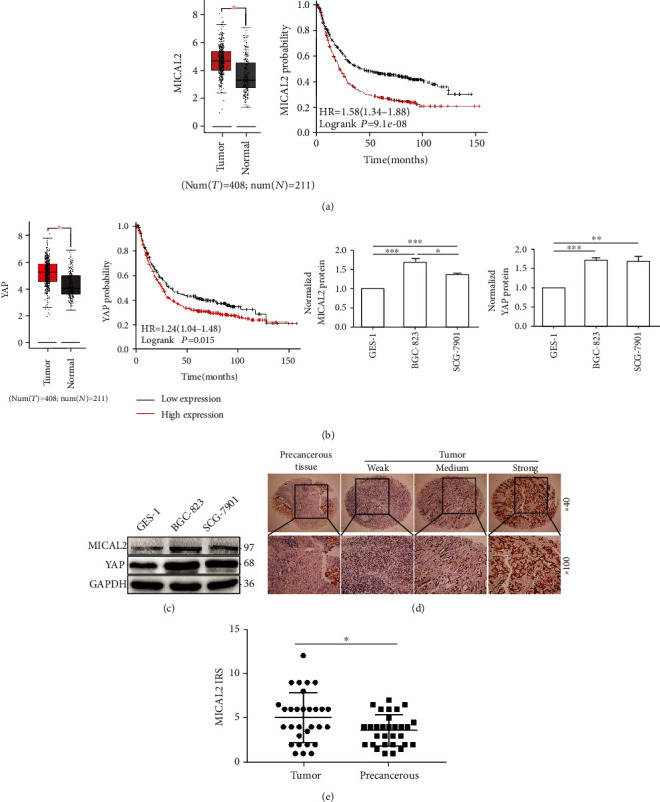
Analysis of MICAL2 levels in gastric cancer tissues. (a) MICAL2 mRNA expression levels are higher in gastric cancer tissues than that in precancerous tissues based on information retrieved from The Cancer Genome Atlas (TCGA) database and the overall survival of patients with gastric cancer showing low or high MICAL2 levels. (b) YAP mRNA expression levels are higher in gastric cancer tissues than that in precancerous tissues and the overall survival of patients with gastric cancer showing low or high YAP levels. (c) MICAL2 and YAP protein levels in GES-1, BGC-823, and SGC-7901 cells. Data in (c) are presented as mean ± SEM of 3 determinations. (d) Representative images of MICAL2 staining in gastric cancer tissues. MICAL2-positive staining is shown in brown, and the nuclei are counterstained with hematoxylin. (e) A scatterplot showing correlations with protein levels in gastric cancer tissue (*n* = 30) and precancerous tissue (*n* = 30) as determined by immunoreactivity scores (IRS). ^∗^*P* < 0.05, ^∗∗^*P* < 0.01, ^∗∗∗^*P* < 0.001.

Gastric cancer-related information obtained from the TCGA database (http://gepia.cancer-pku.cn/) and GTEx data indicated that MICAL2 or YAP mRNA levels were significantly higher in gastric cancer samples compared with those in precancerous tissues (Figures 1(a) and 1(b)). Kaplan–Meier plotter (https://kmplot.com/analysis/)-based analysis also showed that elevated MICAL2 or YAP expression was correlated with shorter overall survival in gastric cancer patients (Figures 1(a) and 1(b)). Next, MICAL2 protein levels were further analyzed by immunoblotting in the gastric cancer cell lines BGC-823 and SGC-7901 and the nonmalignant gastric epithelial cell line GES-1. As shown in Figure 1(c), MICAL2 levels were higher in BGC-823 cells than in SGC-7901 cells and were lowest in GES-1 cells (Figure 1(c)). In addition, YAP levels were higher in both gastric cancer cell lines than in the GES-1 line. MICAL2 protein levels were also analyzed in a tissue microarray containing 30 paired gastric cancer and adjacent nontumor tissues. The immunohistochemical analysis showed that MICAL2 protein levels were significantly higher in gastric cancer tissues than in adjacent normal tissues (*P* < 0.05) (Figures 1(d) and 1(e)). Overall, the *in vitro* and *in vivo* data strongly suggested that MICAL2 is highly expressed in human gastric cancer samples and is associated with poor clinical outcome.

### 3.2. The Effect of MICAL2 on Gastric Cancer Cell Proliferation

**Figure 2:**
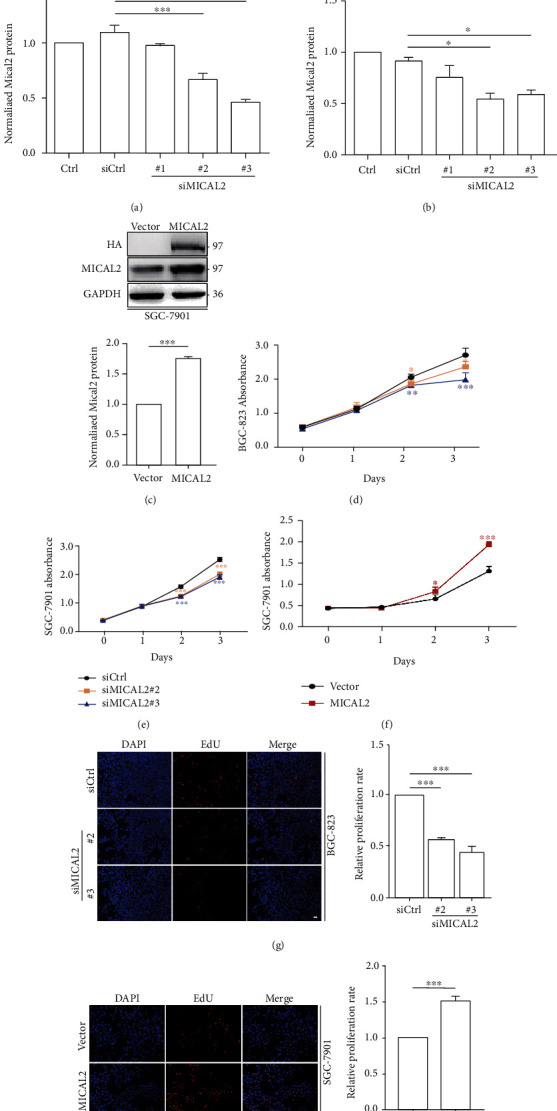
The effect of MICAL2 on the proliferation of gastric cancer cells. (a, b) BGC-823 (a) and SGC-7901 cells (b) were transfected with control siRNA or siRNA specifically targeting MICAL2 (siMICAL2). After 48 h, total protein extracts from cells were analyzed for MICAL2 protein expression. Western blot bands corresponding to MICAL2 were quantified and normalized against GAPDH levels. (c) SGC-7901 cells were transfected with empty vector or MICAL2 overexpression plasmids, and total cellular proteins were extracted and analyzed for MICAL2 expression by Western blotting. Data in (a–c) are presented as mean ± SEM of 3 determinations. ^∗^*P* < 0.05, ^∗∗∗^*P* < 0.001. (d–f) The viability of BGC-823 and SGC-7901 cells transfected with siMICAL2 (d, e) and SGC-7901 cells transfected with MICAL2 overexpression plasmids (f) was assessed by cell counting kit-8 (CCK-8) assay. Data are presented as mean ± SEM of 5 determinations. (d, e) ^∗^*P* < 0.05, ^∗∗^*P* < 0.01, ^∗∗∗^*P* < 0.001 vs. siCtrl. (f) ^∗^*P* < 0.05, ^∗∗∗^*P* < 0.001 vs. the empty vector. (g, h) Representative images of EdU staining in BGC-823 cells transfected with siMICAL2 (g) and SGC-7901 cells transfected with MICAL2 overexpression plasmids (h), and the cell proliferation rate was quantified. Data are presented as mean ± SEM of 5 determinations. Scale bar, 5 *μ*m. ^∗∗∗^*P* < 0.001.

Next, we used both gain- and loss-of-function assays to alter MICAL2 contents and confirm its role in the regulation of gastric cancer cell proliferation. First, we silenced MICAL2 in BGC-823 and SGC-7901 cells using siRNA targeting MICAL2. The knockdown efficiency was determined by Western blotting. As shown in Figures 2(a) and 2(b), siMICAL2 #2 and #3 significantly depleted MICAL2 levels in both BGC-823 and SGC-7901 cells. Additionally, as expected, MICAL2 overexpression markedly increased the MICAL2 protein content (Figure 2(c)). We then also evaluated the effect of MICAL2 on gastric cancer cell proliferation using CCK-8 and EdU staining assays. The results showed that MICAL2 silencing effectively impaired the growth kinetics of BGC-823 and SGC-7901 cells (Figures 2(d), 2(e), and 2(g)), whereas its overexpression enhanced the proliferative ability of SGC-7901 cells (Figures 2(f) and 2(h)). These data indicated that MICAL2 may exert an oncogenic function in gastric cancer.

**Figure 3:**
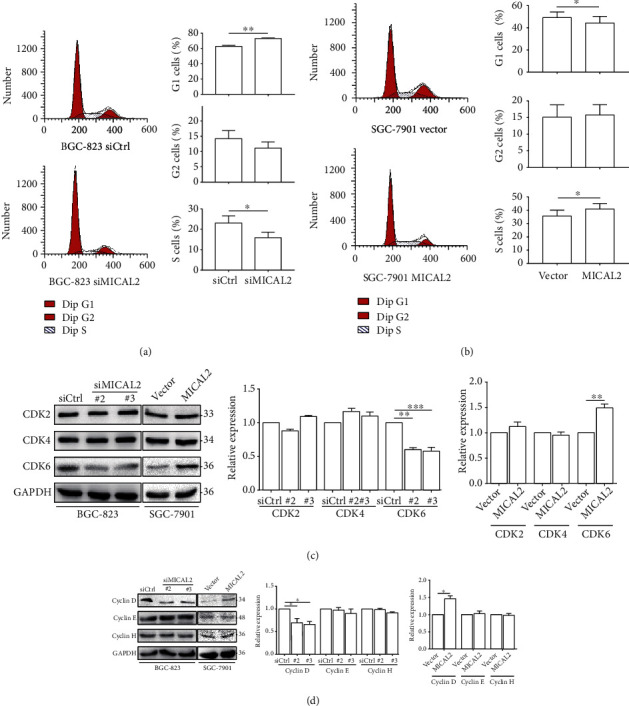
The effect of MICAL2 on the cell cycle. (a, b) Cell cycle progression in BGC-823 (a) and SGC-7901 cells (b) transfected with the indicated siRNA or plasmids was analyzed by flow cytometry. Cell cycle data are shown in histograms. (c, d) MICAL2-depleted BGC-823 cells and MICAL2-overexpressing SGC-7901 cells were subjected to Western blotting analysis for CDK2, CDK4, CDK6 (c), and cyclin D–H (d). Data are presented as mean ± SEM of 3 determinations. ^∗^*P* < 0.05, ^∗∗^*P* < 0.01, ^∗∗∗^*P* < 0.001.

To further understand the potential underlying mechanisms, we examined the effect of MICAL2 on cell cycle progression. For this, BGC-823 cells transfected with siMICAL2 #3 for 48 h, followed by staining with PI, were harvested for analysis of DNA content using flow cytometry. The results showed that the percentage of cells in the S phase was lower in BGC-823 cells than in the controls (Figure 3(a)). In contrast, the percentage of S-phase cells in MICAL2-overexpressing SGC-7901 cells was markedly higher than that of control cells (Figure 3(b)). These findings suggested that MICAL2 plays a role in promoting G1-to-S phase cell cycle progression.

To further confirm that MICAL2 indeed regulates the G1/S-phase transition, gastric cancer cells were treated with siMICAL2 after which the effects on the protein levels of cyclin D and CDK6 were determined by Western blotting analysis. In addition to their roles in regulating G1-to-S-phase progression, cyclin D and CDK6 are also regarded as key targets of the Hippo signaling pathway [28]. The results showed that, compared with the control condition, MICAL2 depletion resulted in a significant decrease in cyclin D and CDK6 protein levels, while MICAL2 overexpression elicited the opposite effect (Figures 3(c) and 3(d)).

### 3.3. MICAL2 Regulates YAP Nuclear Translocation

**Figure 4:**
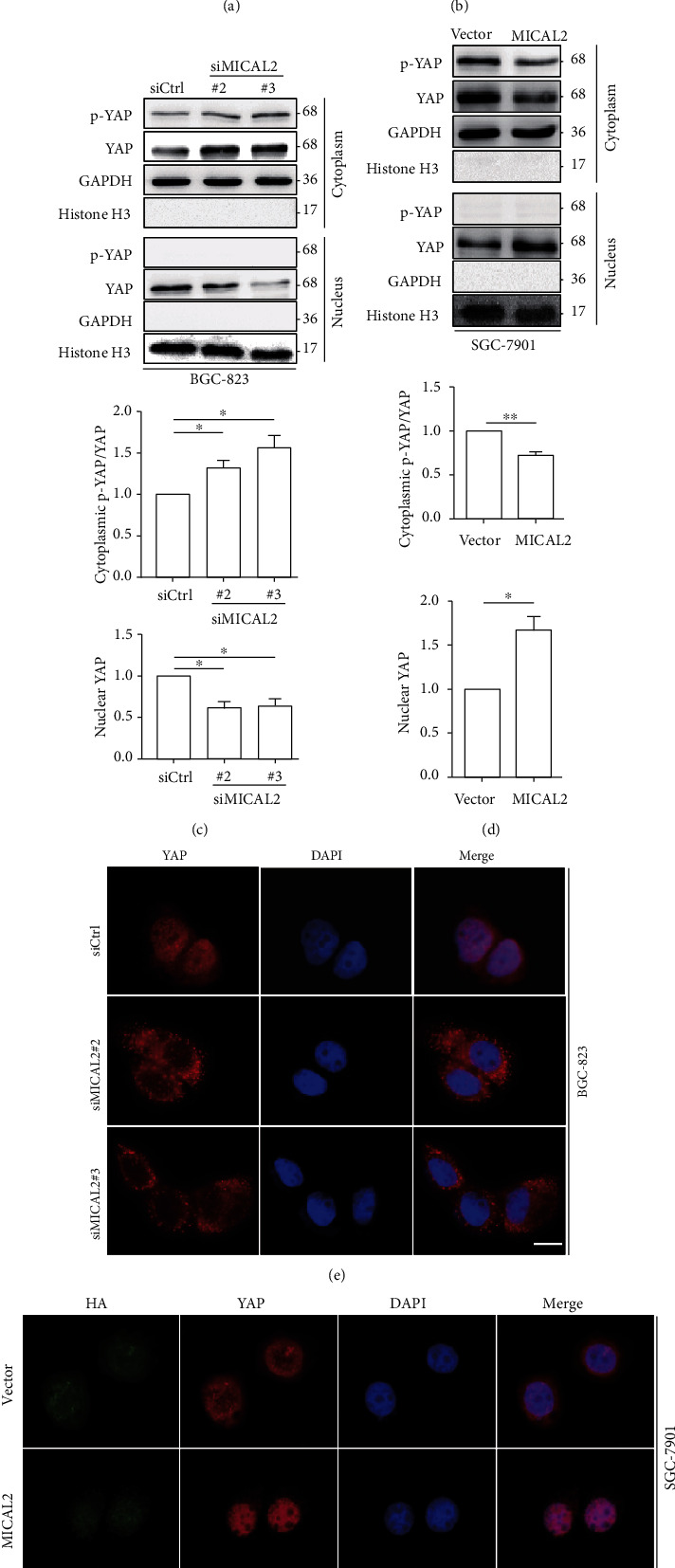
MICAL2 positively regulates YAP protein levels and reduces YAP phosphorylation. (a, b) The protein levels of YAP and p-YAP were detected by Western blotting analysis in BGC-823 cells transfected with siMICAL2 (a) and SGC-7901 cells transfected with MICAL2 overexpression plasmids (b). (c, d) p-YAP/YAP protein levels in cytoplasmic and nuclear extracts of BGC-823 or SGC-7901 cells separately transfected with siMICAL2 (c) or MICAL2 overexpression plasmids (d) were examined. GAPDH served as the cytoplasmic control and histone H3 as the nuclear control. ^∗^*P* < 0.05, ^∗∗^*P* < 0.01, ^∗∗∗^*P* < 0.001. Data are presented as mean ± SEM of 3 determinations. (e, f) Representative immunofluorescence images of YAP staining in BGC-823 cells transfected with siMICAL2 (e) or SGC-7901 cells transfected with MICAL2 overexpression plasmids (f). Scale bar, 5 *μ*m. (g) The viability of MICAL2-silenced BGC-823 cells cotransfected with YAP expression plasmids and (h) MICAL2-overexpressing SGC-7901 cells cotransfected with siYAP#1 was evaluated by cell counting kit-8 (CCK-8) assay. Data are presented as mean ± SEM of 5 determinations. ^∗∗^*P* < 0.01, ^∗∗∗^*P* < 0.001.

We next tested whether MICAL2 modulates YAP expression and subcellular localization in gastric cancer cells. To determine whether MICAL2 regulates YAP expression, we silenced MICAL2 in BGC-823 cells using siMICAL2. As shown in Figure 4(a), the transfection of both siMICAL2 #2 and #3 led to a significant decrease in YAP protein levels. siMICAL2 treatment also increased the p-YAP/YAP ratio in BGC-823 cells. Western blotting analysis further showed significant levels of MICAL2 translocation from the nucleus to the cytoplasm in these cells (Figure 4(c)). As expected, YAP immunofluorescence staining revealed that YAP nuclear content was decreased in MICAL2-silenced BGC-823 cells when compared with that in control cells (Figure 4(e) and Figure S1a). Notably, MICAL2 overexpression only reduced YAP phosphorylation levels and not total YAP content (Figure 4(b)). In contrast, cells overexpressing MICAL2 displayed increased YAP nuclear accumulation as determined by Western blotting and immunofluorescence assays (Figures 4(d) and 4(f) and Figure S1b). These results suggested that the inhibitory effects of MICAL2 on Hippo signaling pathway activation may depend on the promotion of YAP nuclear translocation.

Next, we examined whether YAP mediates MICAL2-induced cell proliferation using both gain- and loss-of-function assays to alter YAP contents (Figures S1c and S1d). We found that MICAL2 overexpression-stimulated gastric cancer cell proliferation was attenuated in siYAP-transfected cells, while YAP overexpression reversed the inhibitory effect of siMICAL2 on cell proliferation (Figures 4(g) and 4(h)). This result indicated that YAP is indeed an effector of MICAL2-mediated regulation of gastric cancer cell proliferation.

### 3.4. MICAL2-Mediated Cell Proliferation Is Independent of the NF-*κ*B and ERK Pathways

Activated NF-*κ*B and ERK signaling pathways play a central role in cell survival and proliferation, and both pathways are involved in G1/S-phase cell cycle transition. To determine whether the effects of MICAL2 on cell proliferation are associated with either the ERK or NF-*κ*B pathway, we assessed the changes in the protein levels of key markers of both pathways in MICAL2-depleted cells. Compared with control cells, neither NF-*κ*B nor p-ERK protein levels were markedly altered with MICAL2 knockdown (Figures S2a and S2c). Furthermore, no significant changes in NF-*κ*B and p-ERK subcellular localization were detected in MICAL2-depleted BGC-823 cells (Figures S2b and S2d). These findings suggested that the effects of MICAL2 on cell proliferation were likely not mediated *via* the NF-*κ*B and ERK signaling pathways.

### 3.5. MICAL2 Promotes YAP Nuclear Translocation through ROS Generation

**Figure 5:**
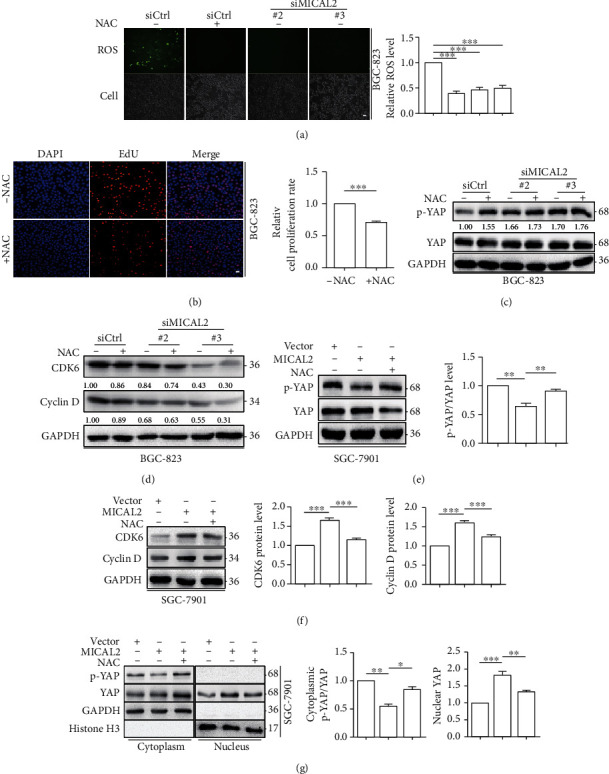
MICAL2 promotes YAP nuclear translocation *via* reactive oxygen species (ROS) generation. (a) The effect of MICAL2 on ROS generation was detected using DCFH-DA in BGC-823 cells transfected with siMICAL2. (b) The effect of N-acetyl-L-cysteine (NAC) on cell proliferation in BGC-823 cells. Scale bar, 5 *μ*m. Data are presented as mean ± SEM of 5 determinations. (c, d) MICAL2-depleted BGC-823 cells were pretreated with NAC, following which the protein levels of p-YAP, YAP (c), CDK6, and cyclin D (d) were detected by Western blotting analysis. (e, f) MICAL2-overexpressing SGC-7901cells were pretreated with NAC, following which the protein levels of p-YAP, YAP (e), CDK6, and cyclin D (f) were quantified. (g) MICAL2-overexpressing SGC-7901 cells were pretreated with NAC after which the protein levels of p-YAP/YAP in cytoplasmic extracts and YAP in nuclear extracts were examined. GAPDH served as the cytoplasmic control and histone H3 as the nuclear control. ^∗^*P* < 0.05, ^∗∗^*P* < 0.01, ^∗∗∗^*P* < 0.001. Data are presented as mean ± SEM of 3 determinations.

Previous study reported that YAP-targeted gene transcription can be regulated by ROS [29]. As MICAL2 participates in ROS production and contributes to YAP nuclear localization, we speculated that MICAL2 might promote YAP nuclear translocation by regulating ROS generation. To test this possibility, we first transfected BGC-823 cells with siMICAL2 (#2 and #3) and examined the effects on ROS generation. As shown in Figure 5(a), ROS levels were suppressed in these cancer cells when compared with those of control cells; a similar effect on cell proliferation was observed when cells were treated with the ROS scavenger NAC and tempol (Figure 5(b) and Figures S3a and S3b).

MICAL2 depletion led to an increase in p-YAP levels and a decrease in those of CDK6 and cyclin D in BGC-823 cells (Figures 5(c) and 5(d) and Figures S3c and S3d). Additionally, NAC and tempol pretreatment did not aggravate these changes greatly, suggesting that ROS production is likely to mediate the effect of MICAL2 on YAP nuclear translocation. Moreover, MICAL2 overexpression in BGC-823 cells resulted in a decrease in p-YAP content and an increase in that of CDK6 and cyclin D, while all these effects were reversed with NAC and tempol pretreatment (Figures 5(e) and 5(f) and Figures S3e and S3f). Next, we treated BGC-823 cells with NAC, tempol, and examined whether YAP subcellular localization was affected. As shown in Figure 5(g) and Figure S3g, NAC and tempol pretreatment prevented YAP nuclear accumulation in MICAL2-overexpressing cells. These results suggested that MICAL2 promotes YAP nuclear localization in a ROS-dependent manner.

### 3.6. MICAL2 Regulates YAP Nuclear Translocation via Cdc42 Activation

**Figure 6:**
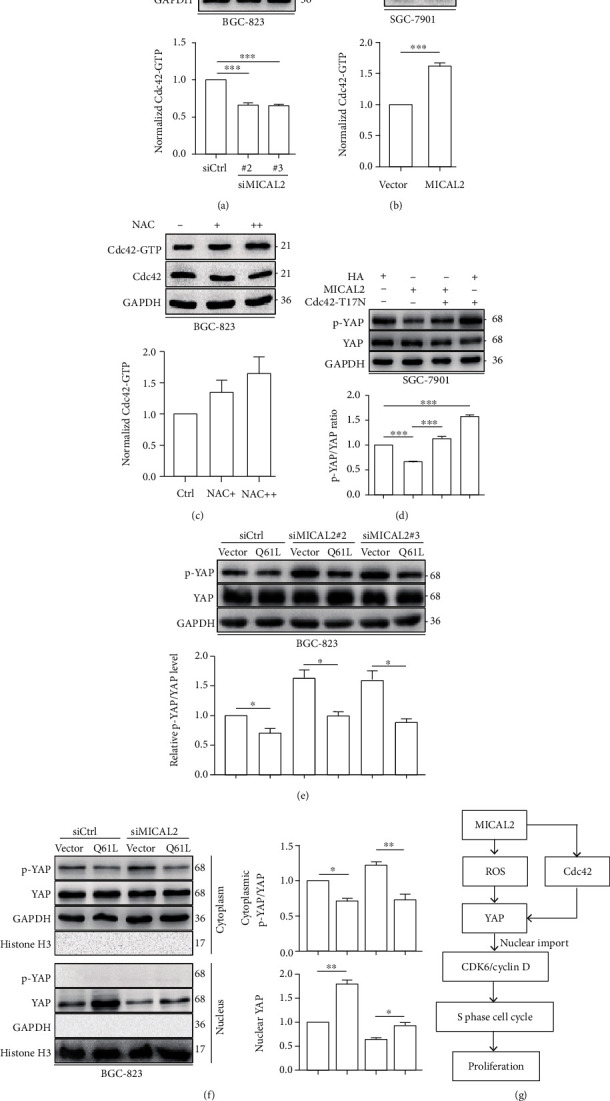
MICAL2 promotes YAP nuclear translocation through Cdc42 activation. (a, b) BGC-823 or SGC-7901 cells were separately transfected with siMICAL2 or MICAL2 overexpression plasmids, following which the levels of the activated form of Cdc42 were measured by pulldown assays. (c) BGC-823 cells were pretreated with 5 mM or 10 mM N-acetyl-L-cysteine (NAC) for 4 h after which the protein levels of GTP-Cdc42 were assessed. (d) MICAL2-depleted BGC-823 cells were transfected with Cdc42-Q61L expression plasmids and analyzed for p-YAP/YAP levels by Western blotting. (e) Cells overexpressing MICAL2 were transfected with Cdc42-T17N and analyzed for p-YAP/YAP levels. (f) MICAL2-depleted BGC-823 cells were transfected with Cdc42-Q61L following which p-YAP/YAP levels in cytoplasmic extracts and YAP levels in nuclear extracts were examined. GAPDH served as the cytoplasmic control and histone H3 as the nuclear control. ^∗^*P* < 0.05, ^∗∗^*P* < 0.01, ^∗∗∗^*P* < 0.001. Data are presented as mean ± SEM of 3 determinations. (g) Schematic model for how MICAL2 regulates YAP nuclear translocation and proliferation in gastric cancer cells.

Finally, we explored whether MICAL2 can promote YAP nuclear translocation by means other than ROS production. Because Cdc42 is known to also increase YAP accumulation in the nucleus and play an important role in the regulation of cell proliferation [5], we assessed whether MICAL2 influenced Cdc42 activation. First, pulldown assays were performed to detect Cdc42 activity in BGC-823 cells transfected with siMICAL2 (#2, #3) and SGC-7901 cells transfected with MICAL2 overexpression plasmids. We found that MICAL2 depletion reduced Cdc42 activation (Figure 6(a)), while the opposite effect was observed when MICAL2 was overexpressed (Figure 6(b)). We further found that treatment with the ROS scavenger NAC and tempol led to only a small increase in Cdc42 activation levels (Figure 6(c) and Figure S4a), suggesting the ROS and Cdc42 independently modulate YAP nuclear translocation. When the Cdc42-T17N (inactive mutant) plasmid was cotransfected with the MICAL2 overexpression plasmid into SGC-7901 cells, the reduced p-YAP/YAP level was reversed (Figure 6(d)). In contrast, the increased p-YAP/YAP level was attenuated when Cdc42-Q61L (active mutant) plasmids were transfected into the MICAL2-depleted BGC-823 cells (Figure 6(e)). In addition, Cdc42-Q61L transfection not only attenuated the upregulation of p-YAP/YAP in the cytoplasm but also reversed the downregulation of YAP in the nucleus of those MICAL2-depleted BGC-823 cells (Figure 6(f)). On the contrary, Cdc42-T17N transfection reversed the downregulation of p-YAP/YAP in the cytoplasm and upregulation of YAP in the nucleus of those MICAL2-overexpressed SGC-7901 cells (Figure S4b). Combined, these findings demonstrated that Cdc42 activation is required for MICAL2-mediated YAP nuclear translocation in gastric cancer cells. A schematic model showing how MICAL2 regulates YAP nuclear translocation and proliferation in gastric cancer cells is shown in Figure 6(g).

## 4. Discussion

It is increasingly clear that cytoskeletal rearrangements can activate oncogenes and induce changes in cell proliferation [30–32]. MICALs are multidomain flavoprotein monooxygenases that catalyze actin redox reactions and destabilize F-actin in cytoskeletal structures [33]. However, to date, relatively few studies have explored the roles of these proteins in the proliferation of human cancer cells. In mammals, the MICAL family of proteins comprises both MICAL (1–3) and MICAL-like (-L1, -L2) forms [34]. In contrast to previous findings indicating that MICAL2 is highly expressed in several other types of cancer [20, 21, 35], in the present study, we found that MICAL2 is highly expressed in gastric cancer cells and is associated with gastric cancer cell proliferation. Subsequently, we investigated the mechanisms by which MICAL2 exerts its proliferative effects in these cancer cells.

Cyclins are cell cycle regulators and function in association with cyclin-dependent kinases (CDKs). Cyclin D, a putative protooncogene, forms a complex with CDK6, thereby accelerating the G1/S cell cycle transition. We showed that CDK6 and cyclin D protein levels were markedly downregulated in MICAL2-knockdown gastric cancer cells, whereas MICAL2 overexpression elicited the opposite effect. Additionally, compared with control cells, the percentage of cells at the S phase of the cell cycle was decreased when MICAL2 was silenced and increased when MICAL2 was overexpressed. Together, these data revealed that MICAL2 promotes cell proliferation *via* stimulating the G1-to-S-phase cell cycle transition, presumably by modulating the levels of CDK6 and cyclin D.

Cyclin D and CDK6 are regulated by several pathways, including the ERK/MAPK, NF-*κ*B, and Hippo signaling pathways. The ERK/MAPK pathway is at the core of the signaling network involved in regulating G1/S-phase progression [36] and both cyclin D and CDK6 are important target genes of this pathway [37, 38]. Although MICAL1, another MICAL family member, was shown to promote ERK phosphorylation and nuclear translocation, a key mechanism mediating breast cancer cell proliferation [39], our results revealed that the knockdown of MICAL2 did not induce significant changes either in p-ERK content or in its nuclear distribution. The NF-*κ*B pathway is also reported to exert its functions in cell cycle progression through the induction of cyclin D during the G1/S-phase transition [40], and the NF-*κ*B subunit p65 has been observed to physically and functionally interact with CDK6 [41]. However, our results showed that MICAL2 depletion did not significantly alter the protein level or subcellular localization of p65. Meanwhile, the Hippo pathway is known to limit organ size, which is associated with its capacity to regulate cell proliferation, apoptosis, and stem cell self-renewal [42, 43]. In this study, we uncovered that YAP is a target of MICAL2, as evidenced by the findings that the knockdown of MICAL2 increased the p-YAP/YAP ratio and inhibited YAP protein levels and its nuclear translocation, opposite to that seen with MICAL2 overexpression. Our results suggested that the function of MICAL2 in gastric cancer cell proliferation is mediated through YAP rather than an ERK- or NF-*κ*B-dependent mechanism.

MICAL family members normally contain an N-terminal flavoprotein monooxygenase (MO) domain; a Lin11, Isl-1, and Mec-3 (LIM) domain; a calponin homology (CH) domain; and a C-terminal coiled-coil (CC) domain [44]. The latter is responsible for an autoinhibitory mechanism that prevents ROS generation [44]. As MICAL2 lacks a CC domain, its MO domain is constitutively active and generates ROS directly. Additionally, MICAL2 oxidizes and promotes depolymerization of F-actin through its MO domain [45]. Consistent with these findings, we showed that ROS contents were markedly reduced in MICAL2-knockdown cells, suggesting that MICAL2 is a primary source of ROS production in gastric cancer cells. The Hippo signaling pathway is particularly sensitive to redox reactions [46, 47]. There is increasing evidence indicating that Hippo signaling activation is critically regulated by oxidative stress. For example, ROS production has been reported to lead to increased YAP mRNA and protein levels in hepatocellular carcinoma cells [48], while Ras activation- and mitochondrial dysfunction-stimulated ROS production can inactivate the Hippo pathway and induce metastatic behavior in benign tumors [49]. Here, we found that the ROS scavenger NAC and antioxidant Tempol prevented MICAL2 overexpression-induced increases in YAP nuclear translocation, CDK6 and cyclin D levels, and cell proliferation and decreases in p-YAP content. These findings provide solid evidence that MICAL2 promotes gastric cancer cell proliferation via ROS-dependent YAP signaling.

It is noteworthy that Cdc42 also associated with YAP nuclear translocation in cancer cells. For example, activated Cdc42 suppresses YAP phosphorylation, resulting in its nuclear localization and, consequently, the growth of, and development of drug resistance in, colorectal cancer cells [50]. Meanwhile, Cdc42 deficiency was shown to induce podocyte apoptosis by inhibiting the activation of the Nwasp/stress fibers/YAP signaling pathway [14]. Studies have demonstrated that ROS can suppress the activation of Cdc42GAP (GTPase-activating protein), a protein that is required for Cdc42-mediated GTP hydrolysis [51], which suggests that ROS might stimulate YAP nuclear translocation via Cdc42 activation. In contrast, our results showed that MICAL2 overexpression resulted in a significant increase in Cdc42 activation in gastric cancer cells. Cdc42 activation reduced YAP phosphorylation levels and increased its nuclear translocation in MICAL2-silenced cells, initially suggestive of a dependency of Cdc42 on MICAL2/YAP signaling; however, our results further revealed that treatment with NAC or tempol resulted in a moderate stimulation of Cdc42 activity, which implied that ROS and Cdc42 activation might serve as independent mediators of MICAL2-induced YAP nuclear translocation. ROS levels can reportedly regulate the expression of MST1, which acts upstream of the Hippo pathway [52], as well as upregulate YAP mRNA and protein levels through the c-Myc pathway in hepatocellular carcinoma cells [48]. The mechanism involved in ROS-mediated regulation of YAP nuclear translocation requires further investigation.

## 5. Conclusion

The present study was a continuation of our previous work in which we demonstrated that the P38/HSP27/cytoskeleton signaling pathway is selectively responsible for MICAL2-induced breast cancer cell invasion [17]. In this study, we have identified a pathway that contributes to MICAL2-mediated gastric cancer cell proliferation involving YAP nuclear translocation. We have also found that MICAL2-mediated cellular ROS generation and Cdc42 activation may independently serve as important mediators of MICAL2-stimulated YAP nuclear translocation. Our results are of importance for understanding the pathophysiological role of MICAL2 in cancer cell proliferation and highlight that targeting MICAL2 may represent a novel intervention strategy for inhibiting gastric cancer progression.

## Data Availability

The data used to support the findings of this study are available from the corresponding author upon request.

## Supplementary Materials

Supplementary Materials Figure S1: the effect of MICAL2 on YAP distribution. (a, b) The effects of MICAL2 on YAP distribution in BGC-823 cells transfected with siMICAL2 (a) or SGC-7901 cells transfected with MICAL2 plasmids (b) were quantified. (c) SGC-7901 cells were transfected with empty vector or YAP overexpression plasmids, and total cellular proteins were extracted and analyzed for YAP expression by Western blotting. (d) BGC-823 cells were transfected with control siRNA or siRNA specifically targeting YAP (siYAP). After 48 h, total protein extracts from cells were analyzed for YAP protein expression. Western blot bands corresponding to YAP, p-YAP, and MICAL2 were quantified and normalized against GAPDH levels. ^∗^*P* < 0.05, ^∗∗^*P* < 0.01. Data are presented as mean ± SEM of 3 determinations. Figure S2: MICAL2 induces cell proliferation independently of the NF-*κ*B and ERK pathways. (a, b) The effects of MICAL2 on NF-*κ*B expression and distribution in BGC-823 cells transfected with siMICAL2 were examined by Western blotting and immunofluorescence assays. (c, d) The effects of MICAL2 on ERK signaling in BGC-823 cells transfected with siMICAL2 were examined by Western blotting and immunofluorescence assays. Scale bar, 5 *μ*m. Figure S3: the effect of tempol on YAP distribution. (a) The effect of tempol on ROS generation was detected using DHE in BGC-823 cells transfected with siMICAL2. (b) The effect of tempol on cell proliferation in BGC-823 cells. Scale bar, 5 *μ*m. Data are presented as mean ± SEM of 5 determinations. (c, d) MICAL2-depleted BGC-823 cells were pretreated with tempol, following which the protein levels of p-YAP, YAP (c), CDK6, and cyclin D (d) were detected by western blotting analysis. (e, f) MICAL2-overexpressing SGC-7901cells were pretreated with tempol, following which the protein levels of p-YAP, YAP (e), CDK6, and cyclin D (f) were quantified. (g) MICAL2-overexpressing SGC-7901 cells were pretreated with tempol after which the protein levels of p-YAP/YAP in cytoplasmic extracts and YAP in nuclear extracts were examined. GAPDH served as the cytoplasmic control and histone H3 as the nuclear control. Data are presented as mean ± SEM of 3 determinations. ^∗^*P* < 0.05, ^∗∗^*P* < 0.01, ^∗∗∗^*P* < 0.001. Figure S4: Cdc42 inactivation prevents MICAL2-induced YAP nuclear translocation. (a) BGC-823 cells were pretreated with 3 mM or 6 mM tempol for 4 h after which the protein levels of GTP-Cdc42 were assessed. (b) MICAL2-overexpressed SGC-7901 cells were transfected with Cdc42-T17N following which p-YAP/YAP levels in cytoplasmic extracts and YAP levels in nuclear extracts were examined. GAPDH served as the cytoplasmic control and histone H3 as the nuclear control. ^∗^*P* < 0.05, ^∗∗^*P* < 0.01. Data are presented as mean ± SEM of 3 determinations.
