# Supplementary material for: MICAL2 Contributes to Gastric Cancer Cell Proliferation by Promoting YAP Dephosphorylation and Nuclear Translocation
Source: Oxid Med Cell Longev. 2021 Oct 5;2021:9955717. doi: 10.1155/2021/9955717 (PMC8510804; doi:10.1155/2021/9955717)
Supplement: Supplementary Materials — Figure S1: the effect of MICAL2 on YAP distribution. (a, b) The effects of MICAL2 on YAP distribution in BGC-823 cells transfected with siMICAL2 (a) or SGC-7901 cells transfected with MICAL2 plasmids (b) were quantified. (c) SGC-7901 cells were transfected with empty vector or YAP overexpression plasmids, and total cellular proteins were extracted and analyzed for YAP expression by Western blotting. (d) BGC-823 cells were transfected with control siRNA or siRNA specifically targeting YAP (siYAP). After 48 h, total protein extracts from cells were analyzed for YAP protein expression. Western blot bands corresponding to YAP, p-YAP, and MICAL2 were quantified and normalized against GAPDH levels. ∗P < 0.05, ∗∗P < 0.01. Data are presented as mean ± SEM of 3 determinations. Figure S2: MICAL2 induces cell proliferation independently of the NF-κB and ERK pathways. (a, b) The effects of MICAL2 on NF-κB expression and distribution in BGC-823 cells transfected with siMICAL2 were examined by Western blotting and immunofluorescence assays. (c, d) The effects of MICAL2 on ERK signaling in BGC-823 cells transfected with siMICAL2 were examined by Western blotting and immunofluorescence assays. Scale bar, 5 μm. Figure S3: the effect of tempol on YAP distribution. (a) The effect of tempol on ROS generation was detected using DHE in BGC-823 cells transfected with siMICAL2. (b) The effect of tempol on cell proliferation in BGC-823 cells. Scale bar, 5 μm. Data are presented as mean ± SEM of 5 determinations. (c, d) MICAL2-depleted BGC-823 cells were pretreated with tempol, following which the protein levels of p-YAP, YAP (c), CDK6, and cyclin D (d) were detected by western blotting analysis. (e, f) MICAL2-overexpressing SGC-7901cells were pretreated with tempol, following which the protein levels of p-YAP, YAP (e), CDK6, and cyclin D (f) were quantified. (g) MICAL2-overexpressing SGC-7901 cells were pretreated with tempol after which the protein levels of p-YAP/YAP in cytoplasmic extrac [file 9955717.f1.docx]

**Supplementary Figure**


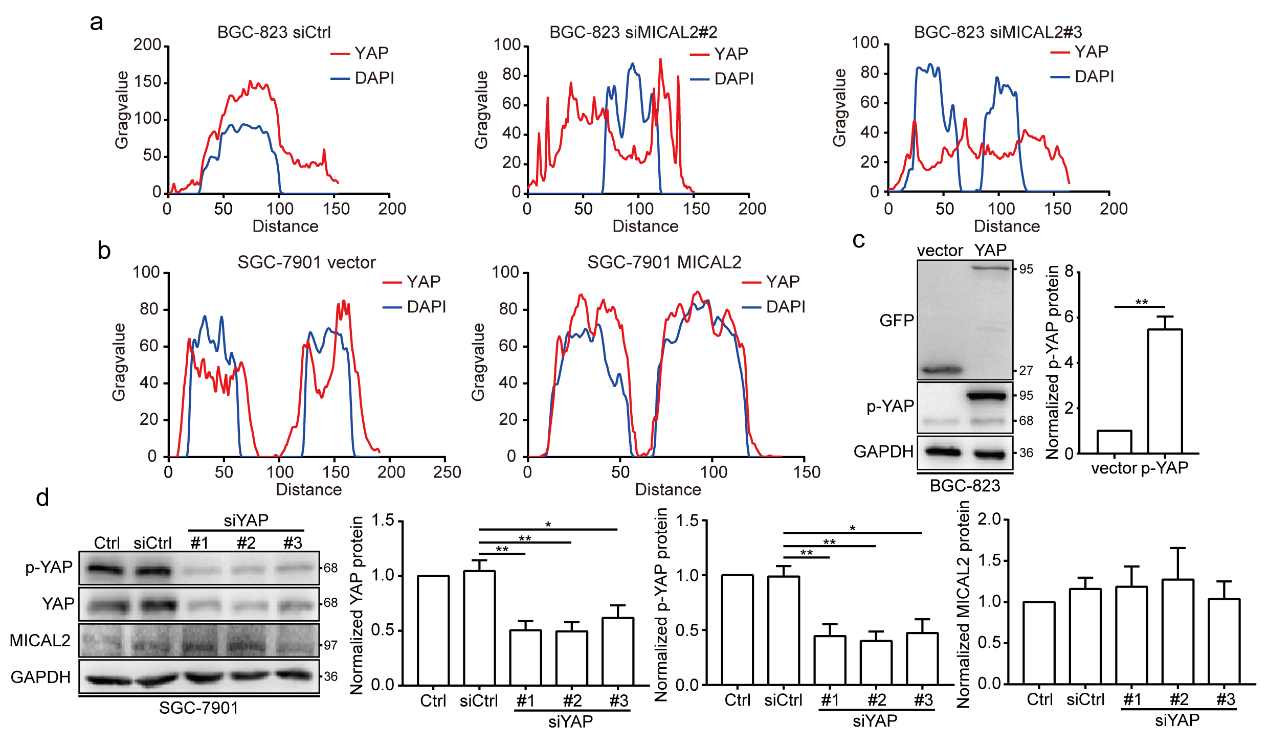


Figure S1: The effect of MICAL2 on YAP distribution. (a, b) The effects of MICAL2 on YAP distribution in BGC-823 cells transfected with siMICAL2 (a) or SGC-7901 cells transfected with MICAL2 plasmids (b) were quantified. (c) SGC-7901 cells were transfected with empty vector or YAP overexpression plasmids and total cellular proteins were extracted and analyzed for YAP expression by western blotting. (d) BGC-823 cells were transfected with control siRNA or siRNA specifically targeting YAP (siYAP). After 48 h, total protein extracts from cells were analyzed for YAP protein expression. Western blot bands corresponding to YAP, p-YAP, MICAL2 were quantified and normalized against GAPDH levels. *: *P*<0.05, **: *P*<0.01. Data are presented as mean ± SEM of 3 determinations.


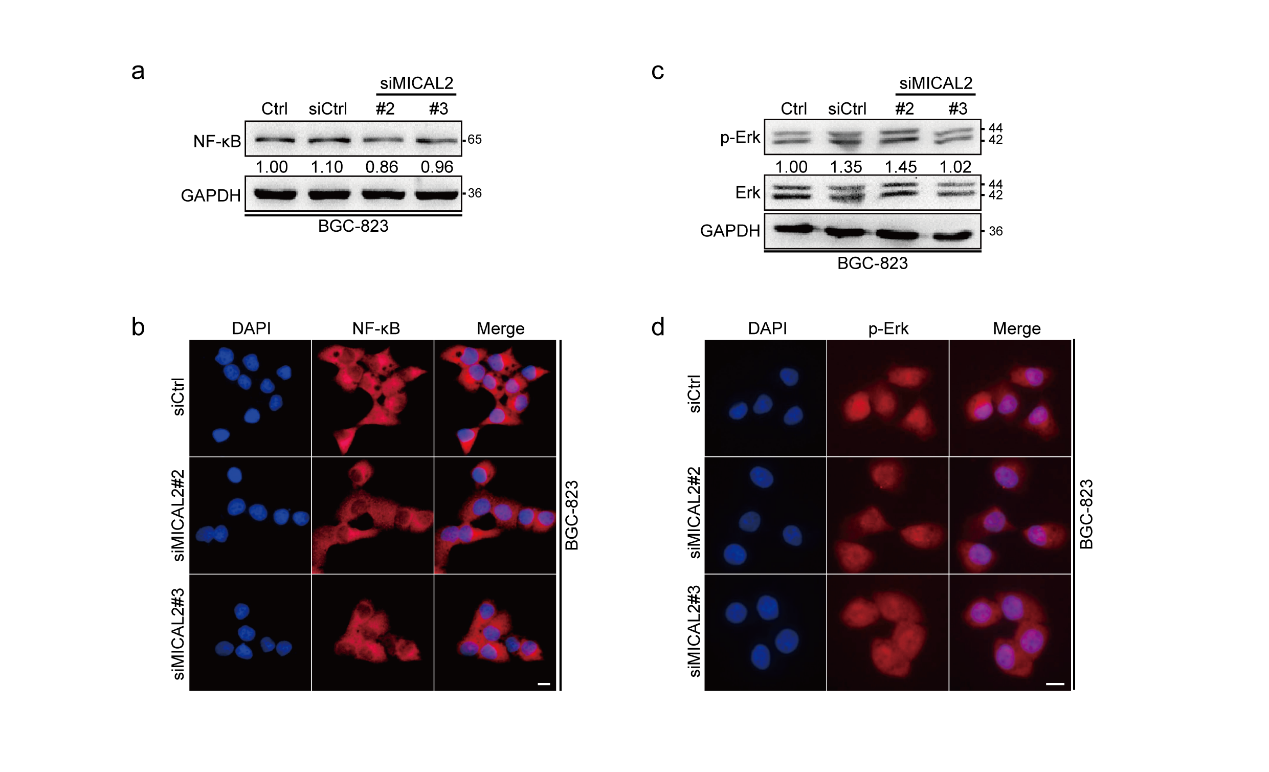


Figure S2: MICAL2 induces cell proliferation independently of the NF-κB and ERK pathways. (a, b) The effects of MICAL2 on NF-κB expression and distribution in BGC-823 cells transfected with siMICAL2 were examined by western blotting and immunofluorescence assays. (c, d) The effects of MICAL2 on ERK signaling in BGC-823 cells transfected with siMICAL2 were examined by western blotting and immunofluorescence assays. Scale bar, 5μm.


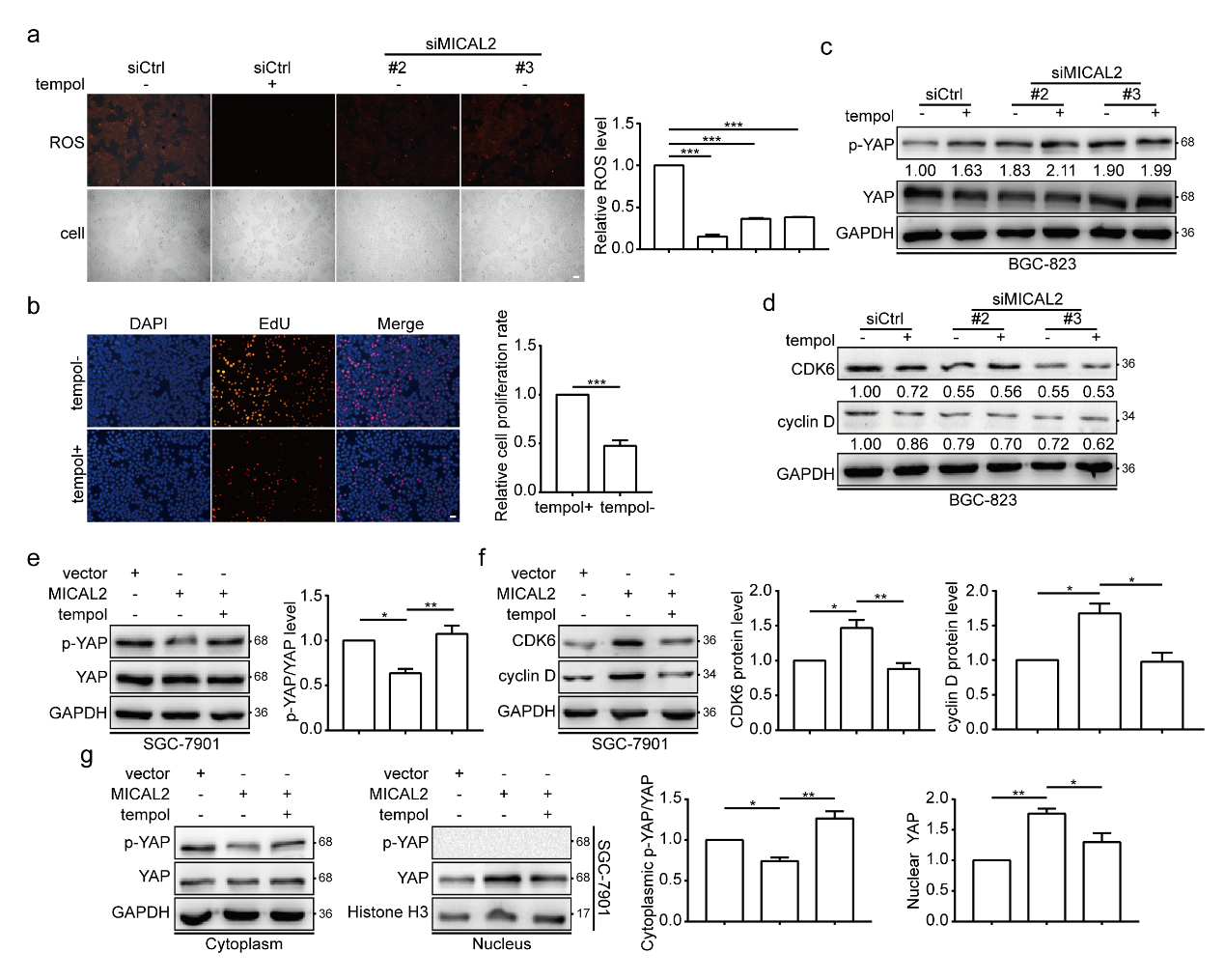


Figure S3: The effect of tempol on YAP distribution. (a) The effect of tempol on ROS generation was detected using DHE in BGC-823 cells transfected with siMICAL2. (b) The effect of tempol on cell proliferation in BGC-823 cells. Scale bar, 5μm. Data are presented as mean ± SEM of 5 determinations. (c, d) MICAL2-depleted BGC-823 cells were pretreated with tempol, following which the protein levels of p-YAP, YAP (c), CDK6, and cyclin D (d) were detected by western blotting analysis. (e, f) MICAL2-overexpressing SGC-7901cells were pretreated with tempol, following which the protein levels of p-YAP, YAP (e), CDK6, and cyclin D (f) were quantified. (g) MICAL2-overexpressing SGC-7901 cells were pretreated with tempol after which the protein levels of p-YAP/YAP in cytoplasmic extracts and YAP in nuclear extracts were examined. GAPDH served as the cytoplasmic control and histone H3 as the nuclear control. Data are presented as mean ± SEM of 3 determinations. *: *P*<0.05, **: *P*<0.01, ***: *P*<0.001.


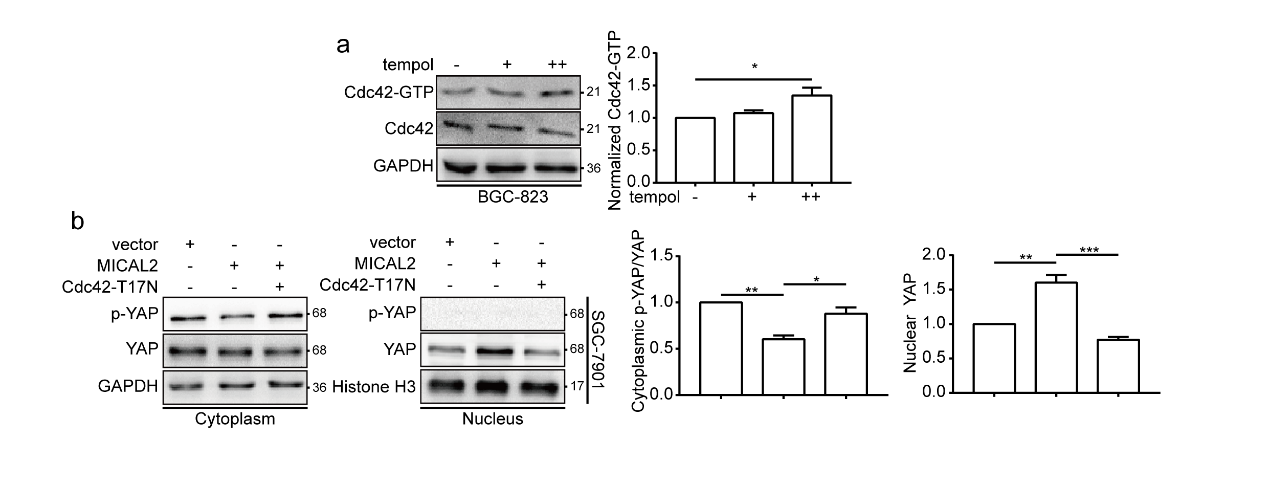


Figure S4: Cdc42 inactivation prevents MICAL2-induced YAP nuclear translocation. (a) BGC-823 cells were pretreated with 3 mM or 6 mM tempol for 4 h after which the protein levels of GTP-Cdc42 were assessed. (b) MICAL2-overexpressed SGC-7901 cells were transfected with Cdc42-T17N following which p-YAP/YAP levels in cytoplasmic extracts and YAP levels in nuclear extracts were examined. GAPDH served as the cytoplasmic control and histone H3 as the nuclear control. *: P<0.05, **: P<0.01. Data are presented as mean ± SEM of 3 determinations.
